# Lung Stereotactic Body Radiotherapy (SBRT): Challenging Scenarios and New Frontiers

**DOI:** 10.3390/jcm14144871

**Published:** 2025-07-09

**Authors:** Serena Badellino, Francesco Cuccia, Marco Galaverni, Marianna Miele, Matteo Sepulcri, Maria Alessia Zerella, Ruggero Spoto, Emanuele Alì, Emanuela Olmetto, Luca Boldrini, Antonio Pontoriero, Paolo Borghetti

**Affiliations:** 1 Radiation Oncology, Department of Oncology, University of Turin, 10125 Turin, Italy; 2 Radiation Oncology, ARNAS Civico Hospital—Palermo, 90145 Palermo, Italy; francesco.cuccia@arnascivico.it; 3 Radiation Oncology Unit, University Hospital of Parma, 43126 Parma, Italy; marco.galaverni@gmail.com; 4 Operative Research Unit of Radiation Oncology, Fondazione Policlinico Universitario Campus Bio-Medico, 00128 Rome, Italy; m.miele@policlinicocampus.it; 5 Radiotherapy, Veneto Institute of Oncology IOV—IRCCS, 35128 Padua, Italy; matteo.sepulcri@iov.veneto.it; 6 Division of Radiotherapy, European Institute of Oncology, Istituto di Ricovero e Cura a Carattere Scientifico, 20141 Milan, Italy; mariaalessia.zerella@ieo.it; 7 Department of Radiotherapy and Radiosurgery, Istituto di Ricovero e Cura a Carattere Scientifico Humanitas Research Hospital, 20089 Milan, Italy; ruggero.spoto@humanitas.it; 8 Radiation Oncology Unit, Azienda USL-Istituto di Ricovero e Cura a Carattere Scientifico Di Reggio Emilia, 42123 Reggio Emilia, Italy; emanuele.ali.06@gmail.com; 9 Radiation Oncology Unit, Oncology Department, Azienda Ospedaliero Universitaria Careggi, Largo GA Brambilla, 50134 Florence, Italy; e.olmetto@gmail.com; 10 Department of Radiology, Radiation Oncology and Hematology, Fondazione Policlinico Universitario A. Gemelli”IRCCS, 00136 Rome, Italy; luca.boldrini@policlinicogemelli.it; 11 Radiation Oncology Unit, Department of Biomedical, Dental and Morphological and Functional Imaging Sciences, University of Messina, 98125 Messina, Italy; apontoriero@unime.it; 12 Radiation Oncology Department, ASST Spedali Civili and University of Brescia, P.le Spedali Civili, 1, 25123 Brescia, Italy; paolobor82@yahoo.it

**Keywords:** stereotactic body radiotherapy, lung tumor, technical aspects

## Abstract

Stereotactic Body Radiotherapy (SBRT) has emerged as a pivotal treatment modality for early-stage non-small cell lung cancer (NSCLC), offering highly precise, high-dose radiation delivery. However, several clinical challenges remain, particularly in the treatment of central or ultracentral tumors, which are located near critical structures such as the heart, bronchi, and great vessels. The introduction of MRI-guided SBRT has significantly improved targeting precision, allowing for better assessment of tumor motion and adjacent organ structures. Additionally, SBRT has demonstrated efficacy in multifocal NSCLC, providing an effective option for patients with multiple primary tumors. Recent advances also highlight the role of SBRT in locally advanced NSCLC, where it is increasingly used as a complementary approach to concurrent chemotherapy or in cases where surgery is not feasible. Moreover, the combination of SBRT with immunotherapy has shown promising potential, enhancing tumor control and immunological responses. Furthermore, SBRTs application in SCLC is gaining momentum as a palliative and potentially curative option for selected patients. This narrative review explores these evolving clinical scenarios, the technical innovations supporting SBRT, and the integration of immunotherapy, providing an in-depth look at the new frontiers of SBRT in lung cancer treatment. Despite the challenges, the ongoing development of personalized approaches and technological advancements continues to push the boundaries of SBRTs clinical utility in lung cancer.

## 1. Introduction

Stereotactic Body Radiotherapy (SBRT) is an effective and well-tolerated treatment for early-stage Non-Small Cell Lung Cancer (NSCLC), representing the first-line non-surgical treatment option for medically inoperable patients [1]. The use of SBRT has increased dramatically in the past 20 years, providing superior local control and survival in comparison with conventionally fractionated radiation, and its indications are rapidly expanding also for lung metastases from different primary sites [2]. Treating high-risk lung cancer patients with radiotherapy is often challenging due to patient characteristics (i.e., target location, lung comorbidities, multiple targets) and technological limits of the delivery units (i.e., suboptimal motion management, need for large treatment margins, difficult target identification), and this may be the cause of treatment-related toxicity both in standard fractionation and SBRT settings. The recent advances in SBRT technology, such as the introduction of magnetic resonance guided radiotherapy (MRIgRT) and optimization of Cone Beam CT imaging (CBCT), further facilitate the safe treatment of patients with anatomically challenging situations. Recent data have highlighted that SBRT may also play a role in the context of locally advanced lung cancer and small cell lung cancer (SCLC), and immune checkpoint inhibitors (ICIs) have significantly improved outcomes in NSCLC, showing promising efficacy in combination with radiation therapy.

**Figure 1:**
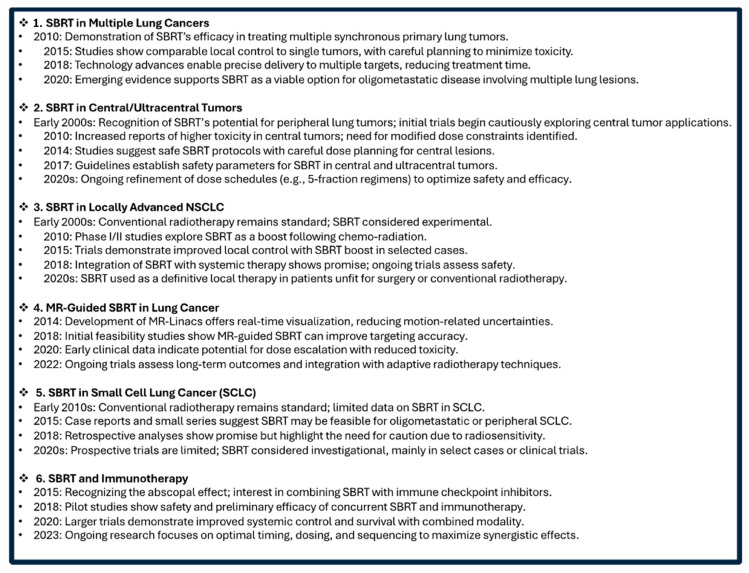
Timeline of SBRT in Challenging Lung Cancer Scenarios.

Figure 1 represents the timeline of the main innovations in SBRT in challenging scenarios.

## 2. Multiple Primary Lung Cancers (MPLCs)

Differentiating multiple primary lung cancers (MPLCs) from intrapulmonary metastasis (IM) remains a significant challenge in cases of multifocal lung cancer. In most patients with multifocal lung tumors, the histological types are often identical, especially in adenocarcinomas, which account for approximately 88% [3,4]. Current diagnostic criteria primarily rely on clinicopathological features [5,6,7]. The first diagnosis of multiple primary lung carcinomas was established at Memorial Sloan Kettering Hospital in 1955. Since then, MPLCs have been categorized into synchronous (sMPLCs) and metachronous (mMPLCs). In 1975, Martini and Melamed [5] proposed criteria to distinguish MPLCs from intrapulmonary metastases.

The 8th Edition of the TNM Classification of Lung Cancer (2016) [8] incorporates clinical and pathological criteria to differentiate second primary tumors from related tumors in cases of sMPLCs and mMPLCs. To enhance clarity, an international expert subcommittee conducted an extensive review of relevant data for this edition [9]. Additionally, the American College of Chest Physicians [6,10,11] incorporated molecular genetic characteristics as diagnostic criteria for both sMPLCs and mMPLCs (see Table 1).

According to the 8th Edition of the TNM [8], multiple lung cancers may be considered as clinical MPLCs if they present different histology on biopsy and as pathological MPLCs if the histological types or comprehensive histologic assessment (CHA) are clearly different, or they are squamous carcinomas that arise from carcinoma in situ.

Accurately identifying MPLCs can be challenging, and these patients are at risk of receiving incorrect treatment based on radiographic and histopathologic staging reports alone [12].

In general, patients with sMPLCs can be offered surgical resection, with 62% 5-year cancer-specific survival. As a result of these favorable outcomes, it is crucial to avoid incorrect staging. Surgeons’ preoperative staging is more accurate than CT and PET-CT. The limitations of preoperative imaging increase the risk of undertreating potentially curable patients [12,13,14].

The advent of genomic mutation testing as a routine clinical method for predictive assessment of NSCLC has marked a major shift in the approach to clonality assessment [15]. Multigene NGS panels have become an increasingly used method in clinical practice, and “comparative molecular profiling” is an emerging approach for determining whether 2 tumors are related (IM) or unrelated (MPLCs).

Surgical resection continues to be the most commonly utilized approach for treating early-stage multiple primary lung cancers (MPLCs) [16,17]. Evidence suggests that performing an anatomical resection of the primary tumor followed by limited resection of co-existing or residual lesions may offer a safer and more advantageous therapeutic strategy [16]. Sub-lobectomy, including wedge resection and segmentectomy, is widely accepted as an alternative option because it preserves a greater amount of pulmonary function, particularly in cases of early-stage lung cancer presenting as ground-glass opacities (GG) [15,17]. However, effective treatment options for high-risk residual lesions post-surgery remain limited, especially for patients unable to undergo further surgical intervention. Previous research indicates that lobectomy is the preferred choice for second primary lung cancers, and sublobar resection does not negatively impact survival for tumors smaller than 2 cm. Conversely, more aggressive surgical procedures such as pneumonectomy are associated with increased comorbidities and may lead to poorer outcomes [16,17].

Spread through air space (STAS) must be taken into consideration in the management of MPLCs as a prognostic factor and therefore in the choice of therapy [15]. The incidence of STAS in diagnosed lung cancer ranges from 15 to 73% based on relevant literature [18,19,20,21]. In meta-analyses, STAS has been demonstrated to be an important adverse prognostic factor for patients receiving surgical removal of lung cancer [22,23,24]. Moreover, considering that patients with MPLCs require a greater extent of lung tissue resection, sub-lobar resection could be considered as the primary surgical approach to better preserve lung function.

Recently, some new approaches, such as SBRT, have eventually been applied to the treatment of sMPLCs. These explorations provided new insights into the comprehensive clinical treatment of MPLCs [25]. Recent case-based studies have reported favorable outcomes of the clinical treatment of residual lesions using ablation and SBRT [26,27]. 

Zhou et al. [25] reported for early-stage sMPLCs cases with lesions with GG or a diameter ≥ 8 mm a high response rate by ablation (CT-guided percutaneous puncture microwave ablation, electromagnetic navigation bronchoscopy-guided microwave ablation) or SBRT; a high response rate was achieved by ablation and SBRT, and 24.5% of the lesions responded to EGFR-TKI treatment. No severe toxicity or adverse event was found. The data demonstrated that ablation treatments have their superiority and safety in the treatment of patients with high-risk residual lesions who were not candidates for surgical resection.

In patients with T1-2N0M0 multiple primary lung cancers (MPLCs), primary tumors classified as T2 stage or presenting as solid lesions exhibited higher rates of tumor recurrence. Additionally, a T2 stage primary tumor was significantly associated with the development of new lesions following initial surgery. Therefore, implementing closer follow-up protocols or adopting more aggressive treatment strategies may be warranted for these patients. Clinical evidence indicates that combining surgery with ablative techniques and/or epidermal growth factor receptor tyrosine kinase inhibitor (EGFR-TKI) therapy demonstrates notable efficacy, superiority, and safety in managing early-stage synchronous MPLCs [25]. Stereotactic body radiation therapy (SBRT) has shown considerable potential in treating primary non-small cell lung cancer (NSCLC) and may serve as a preferable alternative for patients with early-stage sMPLCs where re-operation is not feasible [28] Furthermore, sublobar resection accompanied by limited nodal dissection is recommended for early-stage NSCLC patients at high surgical risk due to low pulmonary reserve or other comorbidities [22,23]. Several studies have reported that patients developing new primary tumors have outcomes comparable to those who do not, suggesting that salvage therapies—including repeated SBRT—are effective for treating subsequent multiple primary MPLCs after initial management of sMPLCs [7,8,9,10,17]. However, studies directly comparing survival outcomes between patients with multiple MPLCs (mMPLCs) and single MPLCs (sMPLCs) have yielded mixed results. Two studies found that patients with mMPLCs experienced improved progression-free survival (PFS) and overall survival (OS) compared to those with sMPLCs [7,9], whereas another study reported similar OS and progression-free rates in patients treated with synchronous SBRT versus those with single-lesion NSCLC [10]. Macagno et al. [29] evaluated the potential for repeat focal treatments, noting excellent tolerability. When the risk of developing additional pulmonary lesions is high and preserving lung function is critical, SBRT or percutaneous thermal ablation (TA) may be preferred. Conversely, when tissue diagnosis is necessary, surgery or TA may be more appropriate for both diagnosis and local control. In cases of synchronous bilateral lesions, a multimodal approach might offer greater benefits. SBRT can be utilized to manage locoregional failures following primary lung cancer treatment or to treat a second primary NSCLC [30,31]. Li et al. reported on a large cohort of patients undergoing multiple courses of definitive-dose lung SBRT, identifying clinical and dosimetric factors associated with radiation-induced lung toxicities (RILT). Factors linked to increased risk of severe RILT included female sex, concurrent SBRT treatments, prior severe RILT, and higher lung V20 values [32]. Overall, multiple courses of lung SBRT have been well tolerated.

## 3. SBRT in Central and Ultracentral Tumors: Is It a Safe Treatment?

The role of SBRT for central and ultracentral tumors still represents a particularly challenging topic and matter of discussion. A phase II study published in 2006 [33] documented high rates of severe toxicity of SBRT for central lung tumors (CT), namely those within two cm of the proximal bronchial tree (PBT), including the distal trachea, main and lobar bronchi up to the first segmental division. Reported 2-year freedom from ≥G3 toxicity was 83% and 54% for peripheral and central tumors, respectively.

Despite the toxicity of SBRT in this “no-fly zone”, the use of SBRT in central tumors spread more and more, thanks to the evolution of radiotherapy techniques (i.e., IMRT, IGRT). A systematic review from 2013 [34] included twenty publications and showed good efficacy, with local control (LC) rates ranging from 64 to 100% at 2–3 years and acceptable rates of toxicity (9% G3-G4 and 3% G5).

Compared to the original definition, a commission of IASLC experts proposed to broaden the concept of central tumors by incorporating all lesions within 2 cm of any critical mediastinal structure in 2015 [35]. A further evolution of the concept of lesion centrality came from the Stanford University group, which documented the feasibility of SBRT in a small retrospective cohort of patients, even where there was direct contact with the bronchial tree, introducing the concept of ultracentral tumors (UT) [36]. The authors did not report cases of toxicity. These encouraging, albeit preliminary, data sparked the enthusiasm of the radiotherapy community, as proved by a systematic review of 10 retrospective studies on UT, published in 2019 [37].

The review confirmed the efficacy of SBRT in this setting, with a 2-year LC rate of 96%, at the price of a cumulative median G3-G4 and G5 toxicity of 10% and 5%, respectively. The first reported cause of G5 toxicity was bronchopulmonary hemorrhage, and high-risk indicators for SBRT-related mortality included PBT Dmax EQD2 ≥ 90 Gy or higher, endobronchial disease, and use of bevacizumab and anticoagulant or antithrombotic drugs.

Data from the first long-awaited prospective study on central lesions (RTOG 0813) were published in 2019 [38].

This was a phase I-II study of dose escalation, which had the aim of identifying the maximum tolerated dose (MTD) of SBRT for CT. In the 100 enrolled patients, toxicity was lower than the prespecified maximum acceptable rate, with 14% at G3-G4 and 5% at G5. Authors conclude that the MTD of SBRT for CT was 12 Gy in 5 fractions, and it was associated with high rates of tumor control, comparable with that of patients with peripheral tumors. Only 17% of treated tumors were ultracentral in RTOG 0813.

The results from a multicenter phase II trial focused on ultracentral tumors (UT) were published in 2021 [39]. The primary endpoint was toxicity and effectiveness of SBRT in tumors within 1 cm of the PBT. Sixty-five patients were treated with an eight-fraction schedule and stratified into group A and group B if the tumor was within 1 cm from the trachea (T)/main bronchi (MB) or >1 cm from T/MB but less than 1 cm from the lobar bronchi, respectively.

Efficacy rates were good, but the survival rate of group A was significantly lower than that of group B due to an excess of G5 toxicity (15% rate), most of which was due to hemoptysis. Tumor distance from T/MB and the dose at 0.2, 0.5, and 1 cc of these structures resulted in being predictive of SBRT-related mortality. The authors concluded that SBRT is unsafe in group A and safe in group B if T/MB received a Dmax < 80 Gy (equivalent dose in 2 Gy fractions, EQD2). A few important limitations were raised to the study by the radiotherapy community: Lobar bronchi were not contoured and therefore not optimized to be protected, and T/MB may have been probably overdosed compared to reported data due to the accepted high PTV Dmax (up to 150%) and the wide margins used for PTV creation (up to 15 mm expansion) [40,41]. In other words, during each fraction at certain moments of the respiratory cycle, T/MB may have moved within isodoses above 100% and up to 150%, resulting in excess fatal toxicity.

In the wake of HILUS results, an updated systematic review and meta-analysis on SBRT in UT from the International Radiosurgery Society (ISRS) was published [42]. Twenty-seven studies were included, and the incidence of severe and fatal toxicities of the previously cited review was substantially confirmed (6% and 4% of G3–G4 and G5 toxicity, respectively). The only prospective included study was the HILUS trial.

The ISRS review was accompanied by recommendations for RT delivery in UT: 60 Gy in 8 fractions, or 60 in 15 fractions when preferring moderate hypofractionation over SBRT, should be prescribed; PTV Dmax should be limited to 150%; antithrombotic/anticoagulant drugs and concomitant targeted or immunotherapies should be discontinued during SBRT or, when impossible, mitigation strategies should be preferred over SBRT (i.e., 60 Gy in 15 fractions), as in cases where intrinsic risk of hemoptysis is high (endobronchial disease).

Following the publication of the ISRS review, results from two additional prospective studies have been released. The LUNG-TECH study, a phase II trial conducted by the EORTC group, evaluated SBRT in centrally located tumors with freedom from local recurrence at 3 years as its primary endpoint [43]. Due to slow patient recruitment, the study was terminated early with 31 patients enrolled out of a planned 150. The prescribed dose was 60 Gy delivered in 8 fractions. At 3 years, local control was observed in 79% of patients, with incidences of ≥G3 and G5 toxicity at 24% and 6%, respectively. The authors concluded that SBRT is a feasible treatment option following comprehensive risk-benefit discussions, noting that despite its efficacy, the risk of significant toxicity remains clinically important. Recent findings from the phase I SUNSET trial have provided optimism regarding the safety and efficacy of SBRT in ultracentral tumors. This multicenter study aimed to determine the maximum tolerated dose (MTD) for ultracentral tumors using the same dose as the LUNG-TECH study. At 2 years, severe toxicity was observed in 6.7% of patients (2 out of 30), while the 3-year local control rate was 90%. Overall, 60 Gy in 8 fractions met the pre-specified criteria for acceptability, with a G3-5 toxicity rate below 30%, and demonstrated excellent local control [44]. The results of the SUNSET trial raised questions regarding the lower observed toxicity compared to the HILUS trial, despite the same prescribed doses. The authors proposed several potential explanations, including the exclusion of endobronchial disease, the implementation of contrast-enhanced simulation CT, thorough delineation of the planning target volume (PTV), the use of narrow margins (5 mm) for PTV definition, and restricting maximum hot-spot doses to ≤120%. Importantly, a higher dose constraint for proton beam therapy (Dmax EQD2 of 141 Gy in SUNSET versus 88.8 Gy in HILUS) appears to be safe when careful planning and delivery are maintained. Nevertheless, the true tolerance of the bronchial tree remains to be fully established. Advances in technology, particularly MRI-guided adaptive radiotherapy (MRIgRT ART), offer promising tools to address this question. These techniques enable real-time, MRI-guided gated delivery that accounts for intrafraction variability, potentially improving safety and precision [45,46].

To date, SBRT in 5–8 fractions is safe for CT without PTV-PBT overlap and more distant than 1 cm from PBT. In UT (with PTV-BPT overlap or nearer than 1 cm to PBT), SBRT in 8–12 fractions is associated with an increased risk of severe toxicity and must be delivered with the aim of prioritizing safety by optimizing dose, fractionation, and contouring; choosing alternative strategies for patients at higher risk of toxicity (i.e., endobronchial disease, anticoagulant drugs); using active motion management techniques in case of respiratory motion > 1 cm (i.e., breath-hold); and limiting hotspots in organs at risk. When this is not possible, the solution in clinical daily practice is to use less hypofractionated regimens (such as 60 Gy in 15 fractions), which are expected to have lower risks of late toxicity but also reduced chances of long-term disease control.

## 4. Stereotactic Body Radiotherapy in Unresectable Locally Advanced Non-Small Cell Lung Cancer

Unresectable stage III NSCLC accounts for 30% of NSCLC diagnoses, and its current standard treatment consists of concurrent chemo-radiotherapy followed by consolidation with Durvalumab in patients with PD-L1 ≥ 1%, with remarkable advantages in terms of progression-free survival (PFS) and overall survival (OS), as reported in the practice-changing PACIFIC trial and validated in several real-world experiences [47,48,49,50]. Nonetheless, despite the undeniable impact of consolidative immunotherapy, intrathoracic and local failures remain a major issue for unresectable stage III disease, leading clinicians to hypothesize new strategies to improve local control with radiotherapy.

The RTOG 0617 trial failed to demonstrate a potential benefit of radiotherapy dose escalation up to 74 Gy, reporting no advantage in terms of local control (LC) and a higher incidence of toxicity compared to the conventional 60 Gy arm [51]. The constant technological progress and the consequent improvement in terms of accuracy for both RT planning and delivery have led clinicians to consider hypofractionation as a means to improve LC rates, and a recent meta-analysis by Mauguen et al. [52] reported an OS advantage when ≥2 Gy/fx are applied, either with sequential or concurrent chemotherapy [53,54].

Recently, a systematic review by Badr Id Said et al. investigated accelerated hypofractionated radiotherapy regimens defined as more than 2 Gy delivered in 10 to 25 fractions in stage III NSCLC, with or without chemotherapy [55]. In a total of 14 trials evaluating definitive accelerated hypofractionation, with a median dose of 60 Gy delivered in a median of 16 fractions, median progression-free survival was 6.4 to 25 months, median survival was 6 to 34 months, and 0% to 8% severe grade ≥3 esophagitis. In 19 studies evaluating accelerated hypofractionated chemoradiation with a median radiation dose of 61.6 Gy delivered in a median of 23 fractions, median progression-free survival was 10 to 25 months, median survival was 13 to 38 months, grade ≥3 esophagitis was 0% to 23.5%, and grade ≥3 pneumonitis was 0% to 11.8%. Only one randomized clinical trial comparing 60 Gy in 15 fractions with 60 Gy in 30 fractions without concurrent chemotherapy did not reveal the superiority of accelerated hypofractionation [56], and the authors of this systematic review suggest that the use of accelerated hypofractionated radiotherapy should be approached with caution, using advanced radiation techniques, especially with concurrent chemotherapy or targeted agents.

Consequently, based on the consolidated role of SBRT in the management of early-stage NSCLC, our scientific community is witnessing a growing interest in the use of stereotactic radiotherapy also for locally advanced disease. The attractiveness of SBRT in this setting relies on a double advantage: From one perspective, very high doses are delivered in a short time with a lower exposure of nearby healthy structures, such as the proximal bronchial tree, esophagus, heart, etc.; secondly, SBRT may play a role as an immuno-booster, enhancing the effect of maintenance Durvalumab [57].

Still, some caveats remain, as locally advanced disease usually involves mediastinal lymph nodes, and the use of SBRT in central and ultra-central disease is potentially related to a higher risk of severe toxicity.

For this purpose, available literature experiences explore different strategies of SBRT for unresectable stage III disease, either proposing SBRT to both primary lesions and positive lymph nodes or combining primary tumor SBRT with conventional mediastinal radiotherapy to mitigate the risk of toxicity. A further strategy reported in the literature is represented using SBRT as a boost to residual disease after conventional chemoradiation.

### 4.1. Definitive SBRT to Primary Tumor and Lymph Nodes

A total of six studies, three retrospective and three prospective, assessed the outcomes of 371 patients with unresectable stage III disease treated with definitive SBRT after chemotherapy. In all these studies, only patients deemed unfit for concurrent chemo-radiation were enrolled.

Literature experiences are summarized in Table 2 [58,59,60,61,62,63]. Most literature experiences reported a preference for 5 fraction schedules with a median total dose of 40 Gy (range, 30–50 Gy) for primary tumor and 30 Gy for nodal disease (range, 25–40 Gy). With a median follow-up of 35 months, acute G ≥ 3 toxicity was observed in 38 cases, including four G5 events, LC rates were quite promising, ranging between 50% and 95.5% at 3 years.

Among these studies, Jia et al. report the largest series of patients treated with SBRT, although including patients with N3 disease treated with induction chemotherapy and subsequent chemo-consolidation after SBRT. Interestingly, the authors reported a significant correlation between BED_10_≥ 85 Gy and OS and PFS, as well as for primary tumor location, with peripheral disease related to improved outcomes compared to central lesions. Nonetheless, as recently highlighted in a literature review by Viani et al., the heterogeneity of the available literature affects the interpretation of the potential benefit of this approach, starting from the total dose, often below the usually recommended threshold of BED_10_ ≥ 100 Gy, and reflecting the divergent planning strategies to cope with the OARs tolerance doses, a factor that might explain the different incidence of severe toxicities among the studies [64].

### 4.2. SBRT to Primary Tumor Plus Conventional Mediastinal Radiotherapy

Several experiences explored the combination of SBRT to the primary tumor and conventional mediastinal RT, as summarized in Table 3 [65,66,67,68,69,70]. The combination of SBRT to the primary lung lesion plus conventional mediastinal chemo-radiation is conceived as a strategy to mitigate the risk of cardio-thoracic toxicity. This approach is reported in six studies (two retrospective, four prospective, including one only available as a protocol) for a total of 130 patients treated. In three studies, SBRT was performed prior to conventional mediastinal treatment; in the remaining, chemo-radiation was anticipated prior to SBRT. The median conventional RT dose was 60 Gy (range, 57–66 Gy), while SBRT was performed in 2–4 fractions for a median total dose of 45 Gy (range, 16–54 Gy). With a median follow-up of 31 months, acute toxicity was mild, with a single G4 event of radiation pneumonitis in the study by Kim et al.; globally, median 2-year LC and OS rates were 85.2% and 70.2%, respectively [66]. Of note, among these studies, the phase II trial by Martel-Lafay et al. was planned in the pre-durvalumab era, and the study was stopped after the PACIFIC trial publication [67]. Recently, Heizerling et al. published a phase II trial designed to evaluate the 1-year PFS rate in subjects with locally advanced NSCLC treated with SBRT followed by concurrent mediastinal chemoradiation with or without consolidation chemotherapy. Sixty-one patients (59 evaluable) were enrolled, with a median follow-up of 29.5 months, with a 1-year PFS of 62.7% (*p* = 0.39, compared with the historical control rate). At the time of the analysis, 37 of 59 evaluable participants were progression-free and alive one year after enrollment (n = 14 progressed, n = 8 died) [70]. Efficacy and safety profiles of this approach were favorable, and these results serve as the basis for the phase 3 study NRG Oncology LU008, which is actually ongoing.

### 4.3. SBRT Boost After Conventional Thoracic Radiotherapy

The use of SBRT as a boost to residual disease after thoracic chemo-radiation is reported in eight studies, enrolling 203 patients. The available literature reports a wide heterogeneity for several features among the studies, making it hard to draw conclusions; starting from residual disease assessment, the use of ^18^FDG-PET is not consistently reported, with five studies not performing metabolic imaging after initial chemo-radiation. Moreover, the definition of residual disease is still controversial, with consequent different target definitions for SBRT (both T and N disease, reported in five studies, vs. only T in three series).

Literature experiences are summarized in Table 4 [71,72,73,74,75,76,77,78].

Significant heterogeneity is also reported on total RT dose for both conventional treatment (median total dose = 49.4 Gy—range; 40–60 Gy) and stereotactic boost (median total dose = 16.5 Gy—range; 13–35 Gy in 2–5 fractions). With a median follow-up of 23.1 months, late G5 toxicity is reported in seven patients, with median 2-year LC and OS rates of 73% and 52.5%, respectively.

Interestingly, in this subgroup, the study recently published by Wu et al. explores the possibility of an accelerated hypofractionated thoracic treatment in 10 fractions (40 Gy/10 fx) followed by 3 levels of SBRT boost up to 35 Gy/5 fx, reporting higher LC rates in the higher dose cohort, still with two G5 events in this arm of the study [78].

## 5. Magnetic Resonance Imaging Guided Radiotherapy (MRIgRT) for “High Risk” Lung Tumors

The recent introduction of magnetic resonance guided radiotherapy (MRIgRT) may improve treatment quality and safety in hard-to-treat categories of patients (i.e., central or ultracentral tumors, lung comorbidities, multiple targets), thanks to innovative technological developments such as direct gating, online treatment plan adaptation, and new techniques for target identification [79,80,81].

Among the advantages expected from the introduction of MRI in the workflow of lung cancer patients undergoing radiotherapy, we can highlight the improved soft tissue contrast, which allows for a more accurate disease volume identification (especially on the mediastinal side), and the possible added value of functional imaging that may disclose new “theragnostic” paradigms successfully integrating the traditional CT- and PET-CT-based nodal diagnostics and driving innovative dose painting protocols with reduced treatment margins [82,83,84].

Besides the advantages related to the aforementioned imaging applications, a second field of theoretical improvement in difficult lung cancer scenarios is the use of online adaptation that reduces as much as possible the unnecessary irradiation of the organs at risk surrounding the target, especially when residual lung tissue should be spared 23 [85].

This application has been explored by Finazzi and colleagues in a cohort of “high-risk” lung cancer patients using 0.35 T MRIgRT online adapted SABR for both primary and secondary lesions. The authors enrolled fifty consecutive patients with a total of 54 lung tumors from May 2016 to November 2018. Most patients had centrally located tumors or other high-risk factors, such as prior lung radiation therapy, previous lung resection, multiple synchronous lung tumors, or coexisting interstitial lung disease. This retrospective analysis, with a median follow-up of 21.7 months, indicates that online-adapted MRIgRT shows encouraging local control rates. No treatment-related deaths occurred, and grade 3 toxicities were observed in only 8% of the patients, an encouraging outcome given the high-risk nature of the patient cohort, which usually experiences severe toxicity within one year [46].

More recently, Eze et al. reported a new clinical pathway for patients with node-positive NSCLC and severely limited pulmonary function, presenting the case of a stage IIB (cN1) NSCLC patient with FEV1 of 38% treated with 48 Gy in 16 fractions that underwent the treatment without toxicity despite the high-risk factors [80].

## 6. Stereotactic Body Radiotherapy in Small Cell Lung Cancer

Small cell lung cancer (SCLC) represents approximately 15% of new diagnoses of thoracic malignancies, and it is characterized by rapid growth, high vascularity, early metastatic spread, significant sensitivity to chemotherapy and radiotherapy, and development of drug resistance during the course of the disease, with a 5-year survival rate of only 7%.

Stage cT1-2N0M0 SCLC only accounts for nearly 5% of patients diagnosed with SCLC, who have a better prognosis, with a 5-year survival rate of up to 50%.

The optimal management of patients with Stage I (cT1-2N0M0) SCLC remains less clear. The National Comprehensive Cancer Network (NCCN) Clinical Practice Guidelines 94 [86] currently recommend lobectomy and mediastinal lymph node dissection, followed by adjuvant chemoradiation therapy (CRT) if node-positive and chemotherapy if node-negative in the case of resectable Stage I SCLC. However, in recent clinical practice guidelines 95 [87], SBRT has also been recommended or, alternatively, chemotherapy with or without conventionally fractionated radiotherapy (RT) for patients who refuse or are not suitable for surgery.

Stereotactic ablative radiation therapy (SABR) is linked to excellent local control rates, patient preference, low toxicity, and cost-effectiveness, making it a safe and effective treatment option for T1-T2N0 small cell lung cancer (SCLC), despite limited evidence from only non-randomized controlled trials [88,89]. In a 2017 multi-institutional study, Verma et al. [90] analyzed 74 patients with inoperable Stage I SCLC treated with SBRT, typically receiving a median dose of 50 Gy over 5 fractions. They reported a high 3-year local control rate of 96%, with minimal toxicity (only 1% experiencing Grade >3 adverse effects). Multivariate analysis indicated that adding chemotherapy improved overall survival (OS) and disease-free survival (DFS), whereas prophylactic cranial irradiation (PCI) did not enhance survival. Most recurrences were distant (45.8%) or nodal (25%). This study concluded that SBRT is a safe, effective local treatment, and chemotherapy provides additional benefit for these patients. Regarding inoperable cT1-T2N0 SCLC, the National Comprehensive Cancer Network (NCCN) recommends chemotherapy with or without conventionally fractionated radiotherapy (RT). However, for patients with poor performance status, conventional RT is not advised. In such cases, SBRT has demonstrated excellent local control and tolerability; even though its control rates are comparable to conventional RT, it is often preferred due to greater convenience, lower toxicity, and better cost profiles. In cases of loco-regional recurrence after SBRT and chemotherapy, chemoradiation may serve as salvage therapy. While prophylactic mediastinal nodal irradiation is a theoretical option, there is no supporting data specifically for cT1-T2N0 SCLC. These outcomes are comparable to those reported in surgical series of patients deemed operable.

Based on these findings, clinical guidelines now incorporate SBRT into the treatment strategy for Stage I SCLC in patients who are not surgical candidates [87].

A subsequent retrospective study by Paximidis et al. compared surgical resection, conventional radiation therapy, and SBRT for Stage I SCLC. They found that surgery offered improved overall survival compared to external beam radiation therapy (EBRT) and SBRT; however, the survival advantage over SBRT was not sustained in patients undergoing limited resection [91]. When patients who underwent surgery were excluded, SBRT resulted in better OS than EBRT. Therefore, for patients ineligible for surgery, SBRT should be considered as an alternative to EBRT. The addition of chemotherapy to any modality further improved survival outcomes and is recommended for suitable candidates.

In a 2021 systematic review and meta-analysis, Safavi et al. summarized outcomes of SBRT for T1-T2N0M0 SCLC, including local control, overall survival, recurrence rates, and toxicity. They identified 11 studies, with 7 (399 patients) included in the meta-analysis. The results showed that the 2-year local control rate exceeded 90%, with 1-year and 2-year OS rates of approximately 86.3% and 63.7%, respectively. Serious toxicities were rare, and most adverse effects were mild, primarily respiratory in nature [92]. While SBRT is effective and safe locally, the higher rates of regional and distant recurrences underscore the metastatic potential of SCLC, highlighting the importance of adjuvant chemotherapy in eligible patients. Another review by Farré et al. discussed the roles of surgery and SBRT in early-stage SCLC. They noted that direct comparisons are challenging due to differences in patient characteristics, such as age and comorbidities, and because surgery generally precedes SBRT in treatment timelines. Although SBRT is a relatively new modality with limited retrospective data, early results are promising. SBRT offers a non-invasive option suitable for elderly patients or those with compromised respiratory function without increasing toxicity. Both surgery and SBRT achieve similar local control rates, though regional relapse may be higher with SBRT—possibly due to mediastinal understaging [93].

In summary, accurate regional and distant staging is crucial for optimizing survival in Stage I SCLC. Given the multiple treatment options, multidisciplinary discussion is essential to determine the most appropriate approach. Current guidelines favor surgical resection—preferably lobectomy with systematic nodal dissection—for well-staged; fit patients. For those inoperable or unfit for surgery, radiation therapy, particularly SBRT, is a safe and effective alternative. All patients should receive systemic therapy if tolerated, due to SCLCs propensity for early metastasis. Additionally, prophylactic cranial irradiation should be considered on a case-by-case basis within a multidisciplinary team.

An emerging scenario is the treatment of oligometastatic SCLC disease. Borghetti et al. published in 2024 a multicenter retrospective analysis of patients with oligometastatic small cell lung cancer (SCLC) treated with stereotactic ablative radiation therapy from 2017 to 2022 [94]. Data from 93 patients were collected, with a total of 132 lesions. Brain and lung metastases were the most frequently treated lesions, accounting for 41.7% and 20.4% of the cases, respectively. Synchronous oligometastatic disease was observed in 23.7% of the patients, metachronous oligometastatic in 35.5%, and oligoprogressive in 40.9% of cases. Median OS was higher for synchronous oligometastasis, and 2-year OS was lower for oligoprogressive SCLC. The median time to next treatment (TnNT) resulted in 14 months for the entire population [94]. Those results suggest that SABR may delay the need for further treatments and may prolong ongoing treatment in oligometastatic SCLC, with the need for prospective studies to confirm these findings.

## 7. Stereotactic Body Radiotherapy and Immunotherapy

Ionizing radiation has the capacity to alter the tumor microenvironment through various mechanisms resulting from radiation-induced cell death. This process can stimulate dendritic cells, thereby promoting the infiltration of lymphocytes into neoplastic cells. Furthermore, radiation modulates the expression of surface receptors essential for lymphocytic infiltration [95]. Stereotactic radiotherapy enhances the immunogenicity of the host, thereby increasing the anti-neoplastic response. This principle underlies the hypothesis of the abscopal effect, characterized by a distant response even in non-irradiated lesions. However, the optimal dosing, fractionation, volumes, and timing between SBRT and immunotherapy remain to be precisely defined [95,96]. Additionally, the expression of PD-L1, patient characteristics, and previous treatments may influence outcomes. The integration of immunotherapy and stereotactic radiotherapy is relevant across all stages of NSCLC.

### 7.1. Neoadjuvant NSCLC

The synergy between immunotherapy and stereotactic radiotherapy in the neoadjuvant setting has been evaluated in a phase II trial that compared patients with early-stage NSCLC receiving immunotherapy with or without SBRT (24 Gy in 3 fractions). The endpoints were safety and pathologic complete response (pCR). Among the 60 patients analyzed, there was a clear advantage in terms of pCR for the SBRT arm, with no significant toxicity reported in relation to the planned surgery [97]. Recently, the authors updated the results, reporting on the prespecified exploratory analysis of DFS in each arm of the trial. For the exploratory endpoint of DFS in each arm of the trial, three-year DFS was 63% (95% CI: 46.0–80.4) in the durvalumab monotherapy arm compared to 67% (95% CI: 49.6–83.4) in the dual therapy arm [98].

Zhao et al. reported the results of the SACTION-1 phase II trial, in which patients with resectable EGFR wild-type stage IIA to IIIB NSCLC were recruited to receive SBRT (24 Gy in 3 daily fractions) to the primary tumor followed by two cycles of PD-1 inhibitor tislelizumab plus platinum-based doublet chemotherapy (Q3W) before surgical resection. Major pathological response (MPR) was observed in 35 (76%, 95% CI 61-87) of 46 patients. The second cycle of immunochemotherapy was withheld in four (9%) patients, and Grade 3 or worse adverse events related to neoadjuvant treatment occurred in 12 patients. There was one treatment-related death, caused by neutropenia. No deaths within 90 days of surgery were reported, showing that preoperative SBRT followed by immunochemotherapy is feasible [99].

Several phase II trials are ongoing to assess the impact of combining radiotherapy with immunotherapy in the neoadjuvant setting.

### 7.2. Early Stage NSCLC

Stereotactic radiotherapy is the primary option for patients who are either inoperable or refuse surgery. There is evidence supporting the high efficacy of radiotherapy with outcomes comparable to those of surgical patients, with minimal side effects [100]. The integration with immunotherapy in this setting evaluates the benefit of adding consolidation treatment to stereotactic radiotherapy, following the principles of the PACIFIC trial in Stage III disease. Recently, a phase II trial compared exclusive ablative SBRT with or without nivolumab for four cycles [101]. The early safety data are promising and suggest potential benefits for the ongoing phase III RCT, though two out of three of these studies were just closed due to failure in efficacy endpoints. We await results from the PACIFIC-4 trial, which has completed accrual.

### 7.3. Oligorecurrent NSCLC

New therapeutic strategies are being explored for patients with oligorecurrent metastatic disease, including the integration of SBRT with immunotherapy. In the PEMBRO-RT trial [102], pembrolizumab was administered after radiotherapy (24 Gy in 3 fractions), while in the MDACC trial [103], radiotherapy (50 Gy in 4 fractions or 45 Gy in 15 fractions) was given concurrently with pembrolizumab.

Both studies were negative for the primary endpoint overall response rate (ORR) and also for progression-free survival (PFS), though the safety of the combination was confirmed.

Subgroup analysis from the MDACC trial suggested a potential advantage for patients with low PD-L1 expression. A recent pooled analysis of the two trials indicates a trend toward benefit for patients treated with SBRT, particularly in terms of overall survival, related to the ability of radiation to stimulate the tumor microenvironment, thus prolonging immunotherapy response [104].

### 7.4. Oligoprogressive NSCLC

Stereotactic radiotherapy can enhance overall survival when added to systemic therapy considered standard of care [105]. Data from the CURB trial [106], which enrolled patients with NSCLC and breast cancer with up to five oligoprogressive lesions, evaluated the benefit of adding SBRT to all sites of progression in addition to standard systemic therapy. CURB demonstrated a benefit in overall survival by adding local treatment to oligoprogressive lesions, with the advantage primarily observed in NSCLC patients. The authors also assessed the disease burden by testing ctDNA before and after radiotherapy, with benefits noted in NSCLC patients. This approach has a strong clinical and practical rationale in order to delay the shift to systemic therapy; moreover, SBRT could be delivered safely to all progressive lesions.

## 8. Future Directions

Stereotactic ablative radiation therapy represents a promising treatment option for patients with various types of lung tumors, particularly in challenging cases due to target location or patients’ characteristics. Ongoing clinical trials and research are essential to refine techniques, delineate patient selection criteria, and enhance the therapeutic window of this innovative approach.

Surgical resection remains the first-choice treatment for MPLCs. Lobectomy and sublobectomy are both acceptable, while pneumonectomy should be avoided. 

Besides surgery, SBRT also appears to be safe and acceptable in the treatment of MPLCs, and no statistical difference in survival, recurrence, and local failure rate is present with respect to the SBRT in single lung cancer. 

Thanks to the initial exploration of the heterogeneity of MPLCs and finally to the use of advanced sequencing technology to clarify the development mechanisms/tumor immune microenvironment, the understanding of MPLCs is now deeper, and it is expected to be even clearer in the near future. 

Recent studies have shown favorable outcomes with SBRT in central/ultracentral lesions, achieving local control similar to peripheral lung tumors, although careful patient selection and imaging guidance are critical. Advanced planning is essential to adhere to dose constraints for organs at risk, and fractional doses may be adjusted to mitigate potential complications, especially thanks to online adaptive strategies. Five to eight fractions of SBRT are currently considered safe in cases without PTV-PBT overlap and when the target is more distant than 1 cm from PBT. In ultracentral tumors, SBRT in 8–12 fractions is associated with an increased risk of severe toxicity and must be delivered with the aim of prioritizing safety; alternative schedules are represented by less hypofractionated regimens. To date, the main issues remain the planning management of central targets and the optimal sparing of healthy nearby structures in order to mitigate toxicity. These factors might explain the large variability in terms of inclusion criteria for tumor size and proximity to the bronchial tree, and consequently the fractionation regimens.

The potential application of SBRT for unresectable stage III NSCLC represents an attractive therapeutic option worthy of investigation. Several approaches are reported in the literature with a wide heterogeneity for results, both in terms of safety and efficacy.

When SBRT is administered as a boost to residual disease, controversies are reported about the definition of residual disease itself, with conflicting evidence about the need for a metabolic imaging re-evaluation after conventional chemoradiation.

Radiation therapy is the therapeutic option in those patients with surgical contraindications, also in SCLC, and SBRT is an emerging technique that appears to be safe and effective for inoperable early-stage cases. Moreover, SBRT may successfully delay the need for further systemic treatments in oligometastatic SCLC, although new prospective studies are needed to confirm these findings.

Incorporating biomarkers such as programmed death-ligand 1 (PD-L1) expression and tumor mutational burden (TMB) can markedly improve the accuracy of predicting the effectiveness of combining stereotactic body radiotherapy (SBRT) with immunotherapy in treating non-small cell lung cancer (NSCLC). PD-L1 expression acts as a key biomarker for pinpointing patients more likely to benefit from immune checkpoint inhibitors, as evidenced by studies like Reck et al. [107], which identified PD-L1 as a predictive indicator for response to pembrolizumab. More recent studies have demonstrated that PD-L1 expression levels are associated with better responses to immune checkpoint inhibitors, and when combined with TMB assessments, they can serve as more reliable predictive biomarkers [108,109]. These biomarkers help identify patients most likely to benefit from combined modality treatments, thereby optimizing therapeutic outcomes and minimizing unnecessary toxicity. Incorporating such molecular profiling into clinical decision-making enhances personalized treatment strategies in NSCLC, especially in the context of evolving immunotherapeutic approaches [110]. As research advances, the integration of PD-L1 and TMB into predictive models holds promise for improving patient selection and treatment efficacy in combined SBRT and immunotherapy regimens [111].

Future studies are advocated to explore the optimal combination with systemic therapies in locally advanced and metastatic NSCLC, which represents an intriguing topic that could significantly impact the treatment landscape for lung cancer, aiming to maximize the immune boost of SBRT.

## 9. Conclusions

The continuous improvements in imaging, motion control, image guidance, and treatment delivery made Stereotactic Body radiotherapy widely disseminated and paved the way to promising results, despite high-quality data often lacking on the use of novel technologies, and more rigorous prospective studies are needed to quantify the observed advantages and better define future treatment guidelines, especially in challenging scenarios.

## Figures and Tables

**Table 1:** Diagnostic Criteria for Multiple Primary Lung Cancers. (**ACCP**, American College of Chest Physicians; **sMPLCs**, synchronous multiple primary lung cancers; **mMPLCs**, metachronous multiple primary lung cancers). Martini Melamed Criteria [5], ACCP Guidelines [6], TNM Staging System (8th Edition) [8].

Martini Melamed Criteria [5]	ACCP Guidelines [6]	TNM Staging System (8th Edition) [8]
**sMPLCs:** Tumors physically distinct and separate Histology: Different histological types Same histological type, but in different segments, lobes, if originating from carcinoma in situ. And no carcinoma in lymphatics common to both. And no extrapulmonary metastases	**sMPLCs:** Different histology Different molecular genetic characteristics Arising from a separate focus of carcinoma in situ Same histology if: Tumors in different lobes And no N2 or N3 involvement And no systemic metastases	**Clinical Criteria for sMPLCs:** Different histological types on biopsy Arguments favoring sMPLCs: Different radiographic appearance or metabolic uptake Different growth rates Different biomarker pattern Absence of lymphatic or systemic metastases
**mMPLCs:** Different histological types Same histological type if: Free interval between cancers ≥2 years Or origin from carcinoma in situ Or second cancer in different lobes and no carcinoma in common lymphatics and extrapulmonary sites	**mMPLCs:** Different histology Different molecular genetic characteristics Arising from a separate focus of carcinoma in situ Same histology, temporarily separated, if: Free interval between cancers ≥4 years And no systemic metastases	**Pathologic criteria for sMPLCs** **(e.g., after resection):** Different histological types Clearly different comprehensive histologic assessment Squamous carcinomas arising from carcinoma in situ. Arguments favoring sMPLCs: Different biomarker pattern Free of lymphatic or systemic metastases.

**Table 2:** Definitive SBRT to primary tumor and lymph nodes in unresectable NSCLC (SBRT, stereotactic body radiotherapy, NSCLC non-small cell lung cancer).

Study (Year)	Study Design	N° of Patients	Median F-Up (Months)	Chemotherapy	SBRT Schedule	Toxicity	LC	OS
Wang et al. (2015) [58]	R	49	25	No	48 Gy/12 fx	No G ≥ 3 events	3 yrs = 95.5%	mOS = 22 mo
Cong et al. (2019) [59]	R	20	17	NR	35 Gy/5 fx	1 G3 RP 2 G5 heart toxicities	1 yr = 61.2%	mOS = 17 mo
Parisi et al. (2019) [60]	P	17	87	Yes (75%)	30 Gy/5 fx for T 25 Gy/5 fx for N	24% late G3 RP	mLPFS = 19.8 mo	mOS = 23 mo
Arcidiacono et al. (2022) [61]	P	50	19	Yes (54%)	45 Gy/5 fx for T 40 Gy/5 fx for N	1 G3 esophageal toxicity	2 yrs = 81% 3 yrs = 50%	2 yrs = 82% 3 yrs = 68%
Kubicek et al. (2022) [62]	P	22	23.1	Yes	50–60 Gy/3–5 fx for T 40–50 Gy/5 fx for N	1 G3 late RP 1 G5 lung toxicity	2 yrs = 81%	mOS = 27.2 mo
Jia et al. (2023) [63]	R	213	40	Yes (67.1%)	35–60 Gy/5–10 fx	9.4% acute G ≥ 3 RP (including one G5) 3.8% acute G ≥ 3 pulmonary hemorrhage	2 yrs = 64.3% 3 yrs = 57.2%	2 yrs = 73.7% 3 yrs = 52%

**Table 3:** SBRT to primary tumor plus conventional mediastinal radiotherapy in unresectable NSCLC (SBRT, stereotactic body radiotherapy; NSCLC, non-small cell lung cancer).

Study (Year)	No. of Patients	Median F-Up (Months)	Mediastinal RT Schedule	SBRT Schedule	Treatment Timing	Toxicity	LC	OS
Chi et al. (2016) [65]	3	23.7	63 Gy/35 fx	40–50 Gy/4 fx	SBRT → Mediastinal chemoradiation	No G ≥ 3 events	2 yrs = 100%	2 yrs = 100%
Kim et al. (2018) [66]	21	12	60 Gy/33 fx	54 Gy/4 fx	Mediastinal chemoradiation → SBRT	1 acute G3 esophagitis 2 acute G ≥ 3 RP	2 yrs = 74.2%	2 yrs = 60.5%
Martel-Lafay et al. (2021) [67]	25	58	66 Gy/33 fx	54 Gy/3 fx	Mediastinal chemoradiation → SBRT	1 acute G3 RP	6 mo = 79%	mOS = 51.6 mo
Williams et al. (2024) [68]	21	NR	60 Gy/30 fx	12–16 Gy/2 fx	SBRT → Mediastinal chemoradiation	1 acute G4 RP	2 yrs = 81.6%	2 yrs = 50.3%
Coutu et al. (2024) [69]	NR	NR	57.8–60 Gy/30 fx	13–20 Gy/2 fx	Mediastinal chemoradiation → SBRT (plus durvalumab)	NR	NR	NR
Heinzerling et al. (2025) [70]	61	29.5	60 Gy/30 fx	50–54 Gy/3–5 fx	SBRT → Mediastinal chemoradiation	2 G3 RP 1 G3 esophagitis 5 G5 (dyspnea, RP, respiratory failure)	NR	NR

**Table 4:** SBRT boost after conventional thoracic radiotherapy in unresectable NSCLC (SBRT, stereotactic body radiotherapy; NSCLC, non-small cell lung cancer).

Study (Year)	No. of Patients	Median F-Up (Months)	CT-RT Schedule	Time Interval CRT-SBRT	SBRT Schedule	Toxicity	LC	OS
Salazar et al. (2008) [71]	30	44	45 Gy/25 fx (no chemo)	NR	22.5 Gy/3 fx	No G ≥ 3 events	73% stage IIIA; 47% stage IIIB	mOS = 15 mo
Feddock et al. (2013) [72]	35	13	59.4 Gy/33 fx	2 mo	20 Gy/2 fx or 19.5 Gy/3 fx	5 G3 RP 2 G5 hemorrhages	82.9%	NR
Karam et al. (2013) [73]	16	14	50.4 Gy/28 fx	20 days	4–6 Gy/5 fx	No G ≥ 3 events	1 yr = 76%	1 yr = 78%
Hepel et al. (2016) [74]	12	15.5	50.4 Gy	7–30 days	8–14 Gy/2 fx	1 G5 hemoptysis	1 yr = 78%	1 yr = 67%
Kumar et al. (2017) [75]	37	25.2	60 Gy	4 mo	10 Gy/2 fx or 6.5 Gy/3 fx	13.5% G3 RP 5.4% G5 hemorrhage	78%	mOS = 25 mo
Higgins et al. (2017) [76]	19	13	44 Gy	NR	9–10 Gy/2 fx or 5–6 Gy/5 fx	10.5% G5 pulmonary and esophageal toxicities	3 yrs = 56%	3 yrs = 39%
Doyen et al. (2018) [77]	26	37.1	46 Gy/23 fx	21 days	21–36 Gy/3 fx	1 G4 fistula 1 G5 hemoptysis	2 yrs = 70.3%	2 yrs = 50.8%
Wu et al. (2024) [78]	28	NR	40 Gy/10 fx	NR	25–35 Gy/5 fx	11% acute G ≥ 3 events 7% late G ≥ 3 events	2 yrs = 74%, 85.7%, 100% (dose levels)	2 yrs = 30%, 76.2%, 55.6%
